# The induction effect of hydroxyurea and metformin on fetal globin in the K562 cell line

**DOI:** 10.1186/s10020-025-01184-8

**Published:** 2025-04-08

**Authors:** Mohammad Eini, Hossain Safarpour, Ebrahim Miri-Moghddam

**Affiliations:** 1 Department of Hematology, Faculty of paramedical, Birjand University of Medical Science, Birjand, Iran; 2 Cellular & Molecular Research Center, Birjand University of Medical Science, Birjand, Iran; 3 Department of Molecular Medicine, Faculty of Medicine, Cardiovascular Diseases Research Center, Birjand University of Medical Science, Birjand, Iran

**Keywords:** Beta-thalassemia, Hemoglobin F, Hydroxyurea, Metformin, *IGF2BP1*, *GCNT2*

## Abstract

Despite the established efficacy of hydroxyurea (HU) in increasing fetal hemoglobin (Hb F) levels in patients with intermedia beta-thalassemia (β-thal) and sickle cell anemia, the precise molecular mechanisms underlying these effects remain largely elusive. Understanding these mechanisms is paramount for identifying alternative therapeutic approaches to increase Hb F production while minimizing adverse effects. In this study, we employed weighted gene co-expression network analysis (WGCNA) to investigate the molecular underpinnings of γ-globin switching within GSE90878 dataset. Leveraging this information, we aimed to predict the transcriptome network and elucidate the mechanism of action of HU and Metformin (Met) on this network comprehensively. Through bioinformatic analysis, we identified IGF2BP1 and GCNT2 as key regulators of the γ-globin switching mechanism. To experimentally validate these findings, we utilized the K562 cell line as an erythroid model. Cells were treated with HU (50, 100, and 150 µM) and Met (50, 100, and 150 µM) for 24, 48, and 72 h. The expression levels of the *GCNT2*, *γ-globin*, *IGF2BP1*, miR-199a/b-5p, miR-451-5p and miR-144-3p were quantified using real-time polymerase chain reaction (qPCR). Our results revealed that treatment with HU (150 µM), Met (100 µM), and combination of HU-Met (150/100 µM) significantly increased *IGF2BP1* expression by 6.2, 5.3, and 7.1-fold, respectively, after 24 h treatment. Furthermore, treatment with HU (50 µM), Met (50 µM) and HU/Met (50/50 µM) for 24 h led to a 3.3, 1.2, and 5-fold decrease in *GCNT2* gene expression, respectively. Notably, the highest levels of γ-globin expression and Hb F production were observed with HU (100 µM), Met (50 µM), and HU/Met (100/50 µM). This study provides compelling evidence that HU and Met significantly enhance γ-globin expression and Hb F production in the K562 cell line. Our findings suggest that these drugs exert their effects by modulating the expression of *IGF2BP1* and *GCNT2*, thus offering valuable insights into potential therapeutic strategies for disorders characterized by low Hb F levels.

**Graphic abstract:**

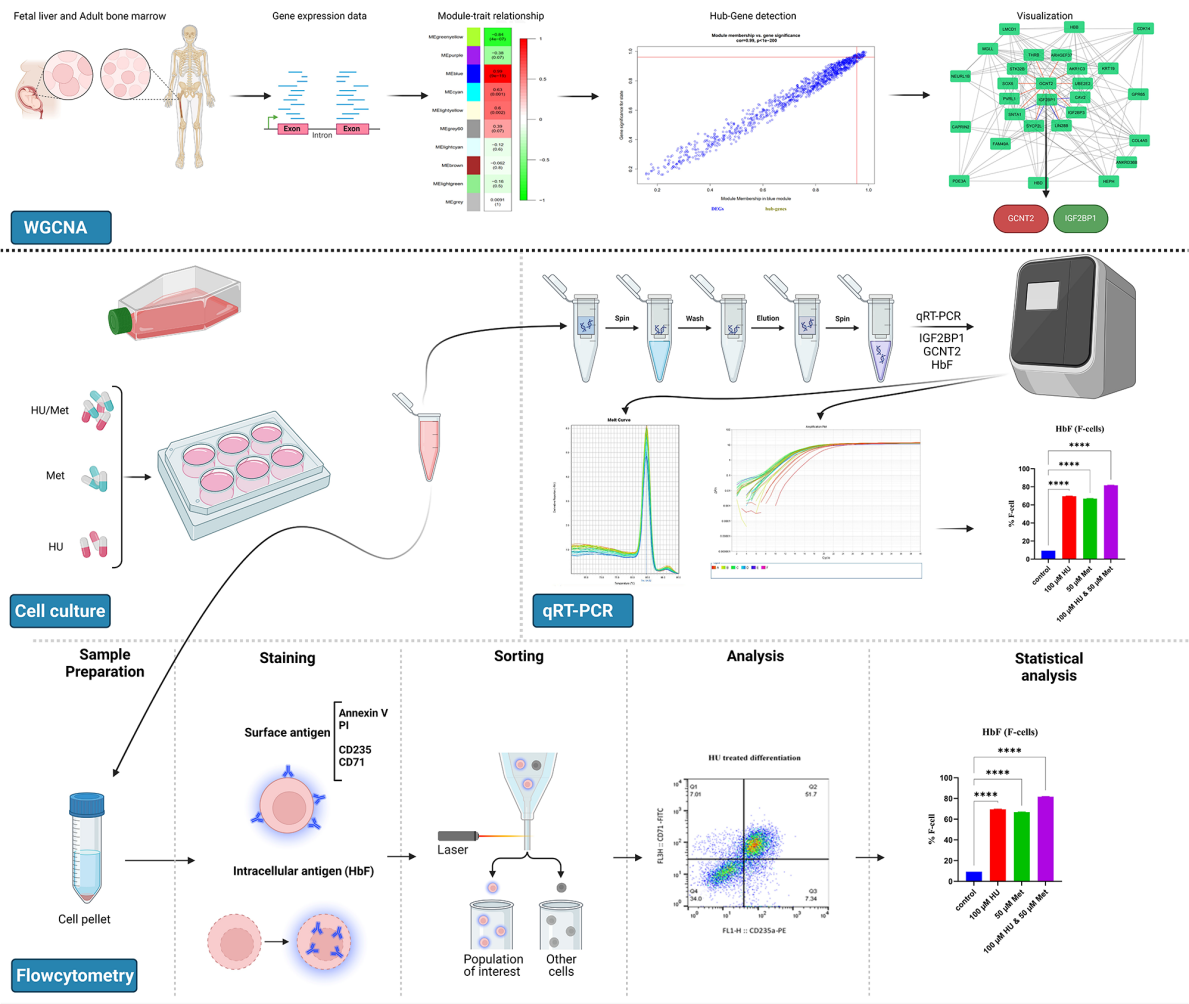

**Supplementary Information:**

The online version contains supplementary material available at 10.1186/s10020-025-01184-8.

## Introduction

Beta-thalassemia (β-thal) is a prevalent genetic disorder affecting approximately 1.5% of newborns globally, translating to an estimated 50,000-100,000 new cases annually (Liu et al. 2018). In β-thal, mutations within the *β-globin* gene results in reduced or absent β-globin chain production, leading to an excess of ɑ-chains. This imbalance in globin chain synthesis disrupts erythropoiesis, resulting in effective red blood cell production (Galanello and Origa 2010). Patients with β-thal are typically classified into three categories: major, intermediate, and minor based on the severity of their clinical manifestation. Regular red blood cell transfusion constitutes the primary for major β-thal, aiming to maintain adequate hemoglobin levels and improv overall quality of life. However, each unit of transfused blood introduces a substantial amount of iron into the bloodstream (approximately 300 mg) leading to iron overload and the risk of organs damage. To mitigate these adverse effects of iron overload, iron chelation therapy administered. Iron chelators facilitate the removal of excess iron from the body through urine and feces. However, it is important to note that some iron chelators may have potential to chelate iron within the central nervous system, potentially impairing neurological development and contributing to cognitive deficits (Belmont and Kwiatkowski 2017; Conrad and Proneth 2019; Taher and Saliba 2017).

Sickle cell anemia is a hereditary hemoglobinopathy characterized by the production of abnormal hemoglobin S (Hb S). Under conditions of low oxygen tension, Hb S polymerizes, causing red blood cells to assume a rigid, sickle shape. This pathological alteration lead to hemolytic anemia, vaso-occlusive crises, and a spectrum of complications affecting multiple organ systems (Kavanagh et al. 2022). Sickle cell anemia is inherited in an autosomal recessive manner. Its prevalence is particularly high in populations of African descent and in regions endemic to malaria, where the heterozygous sickle cell trait confers a selective advantage against malaria infection (Das and Talukder 2002). Current management strategies for sickle cell anemia include HU therapy, blood transfusions, and supportive care aimed at alleviating symptoms and minimizing complications (Claster and Vichinsky 2003).

Hemoglobin F (Hb F) reactivation represents a promising therapeutic approach for hemoglobinopathies, involving the reprogramming of erythroid precursors in adult patients. Pharmacologic agents such as HU, decitabine, metformin (Met) and short-chain fatty acids (SCFAs) have shown potential in inducing Hb F reactivation (Koshy et al. 2000; Liakopoulou et al. 2011). While the precise molecular mechanism and transcription network underlying γ-globin gene switching during HU treatment remain unclear, epigenetic regulation plays a pivotal role. This includes alterations in chromosome structure, higher-order chromatin organization, and modifications in histone and DNA methylation (Sankaran et al. 2010). Key transcription factors involved in γ-globin gene switching include *BCL11A*,* SOX6*,* KLF1*,* ZFPM1*,* LSD1*,* LDB1*,* DNMT1*, and *FOXO3*, all of which are crucial for the regulation of fetal hemoglobin expression (Liu et al. 2018; Gilmartin et al. 2021; Krivega et al. 2015; Sangerman et al. 2006; Sankaran 2011; Shi et al. 2013; Xu et al. 2010; Zhou et al. 2010).

HU exerts its antineoplastic effects by intracellular metabolism into nitric oxide (NO). Elevated cytosolic NO levels lead to an increase in cyclic guanosine monophosphate (cGMP) and protein kinase G (PKG) activity. This cascade upregulate phosphodiesterase 2 (PDE2), reactive oxygen species (ROS), and p38 MAPK while inhibiting Bcl-2-associated death promoter (BAD) and nuclear factor kappa B inhibitor 2 (NFKBI2). ROS can activate the mitogen-activated protein kinase 1/2 (MAPK1/2) signaling pathway, upregulate nuclear factor erythroid 2–related factor 2 (NFE2L2), and stimulate the expression of small MAF proteins (MAFF, MAFG, MAFK). Ultimately, this complex signaling cascade contributes to the activation of specific protein 1 (SP1) and cyclin-dependent kinase inhibitor 2B (CDKN2B) genes, potentially through the MAPK8 pathway. Additionally, studies have demonstrated that HU can inhibit *BCL11A* and enhance γ-globin expression through the inhibition of MYC (Pule et al. 2015). Met is an antidiabetic drug approved for the treatment of type 2 diabetes mellitus in the United States. Its primary mechanism of action involves suppressing hepatic gluconeogenesis. While the insulin-sensitizing effects of Met in peripheral tissues such as muscle and adipose tissue, remain a subject of ongoing investigation, it has been shown to induce γ-globin expression in erythroid precursors by modulating the FOXO3-pathway, without significantly affecting the expression of *BCL11A*, *MYB*, or *KLF1* (Zhang et al. 2017).

MicroRNAs (miRNA) are short non-coding RNA molecules, typically 21–25 nucleotides in length, that exhibit remarkable evolutionary conservation. They play critical roles in diverse biological processes, including cell fate determination, proliferation, differentiation, and the pathogenesis of cancer. Several miRNAs have been implicated in the regulation of globin gene expression, specifically in the reactivation of γ-globin gene expression, a key therapeutic target for diseases like β-thal. Notably, miR-451-5p, miR-144-3p and miR-199 a/b-5p are believed to regulate various aspects of erythroid cells function, including early erythroid cell maturation and proliferation, γ-globin expression, and enucleation (Sankaran et al. 2010). Precisely determining the expression levels of these microRNAs is critical for identifying the regulatory network in *γ-globin* gene expression and for the development of novel therapeutic strategies aimed at inducing γ-globin production.

Our understanding of the complex regulatory mechanisms underlying γ-globin gene switching remains incomplete. To gain a comprehensive understanding of these processes, we employed Weighted Gene Co-expression Network Analysis (WGCNA), a systems biology approach that enables the identification of previously unrecognized gene interactions and pathways. This study method has helped identify previously unrecognized gene interactions and pathways involved in γ-globin switching. This study analyzed mRNA expression profiles from both fetal and adult liver tissues to identify differentially expressed genes associated with γ-globin gene switching. By applying WGCNA, we identified *IGF2BP1* as an upregulated gene and *GCNT2* as a downregulated gene in fetal liver cells compared to adult liver cells.

Given the intricate nature of the mechanisms by which HU and Met induce γ-globin expression, we plan to further investigate the roles of the selected genes *GCNT2* and *IGF2BP3*, along with key upregulated and downregulated miRNAs (miR-451-5p, miR-144-3p, and miR-199a/b-5p), in modulating γ-globin expression in K562 cells treated with HU and Met. This research will contribute to a deeper understanding of the molecular mechanisms underlying γ-globin gene switching and may identify novel therapeutic targets for β-globinopathies.

## Materials and methods

### β-thal gene expression dataset and transcriptome analysis

**Fig. 1:**
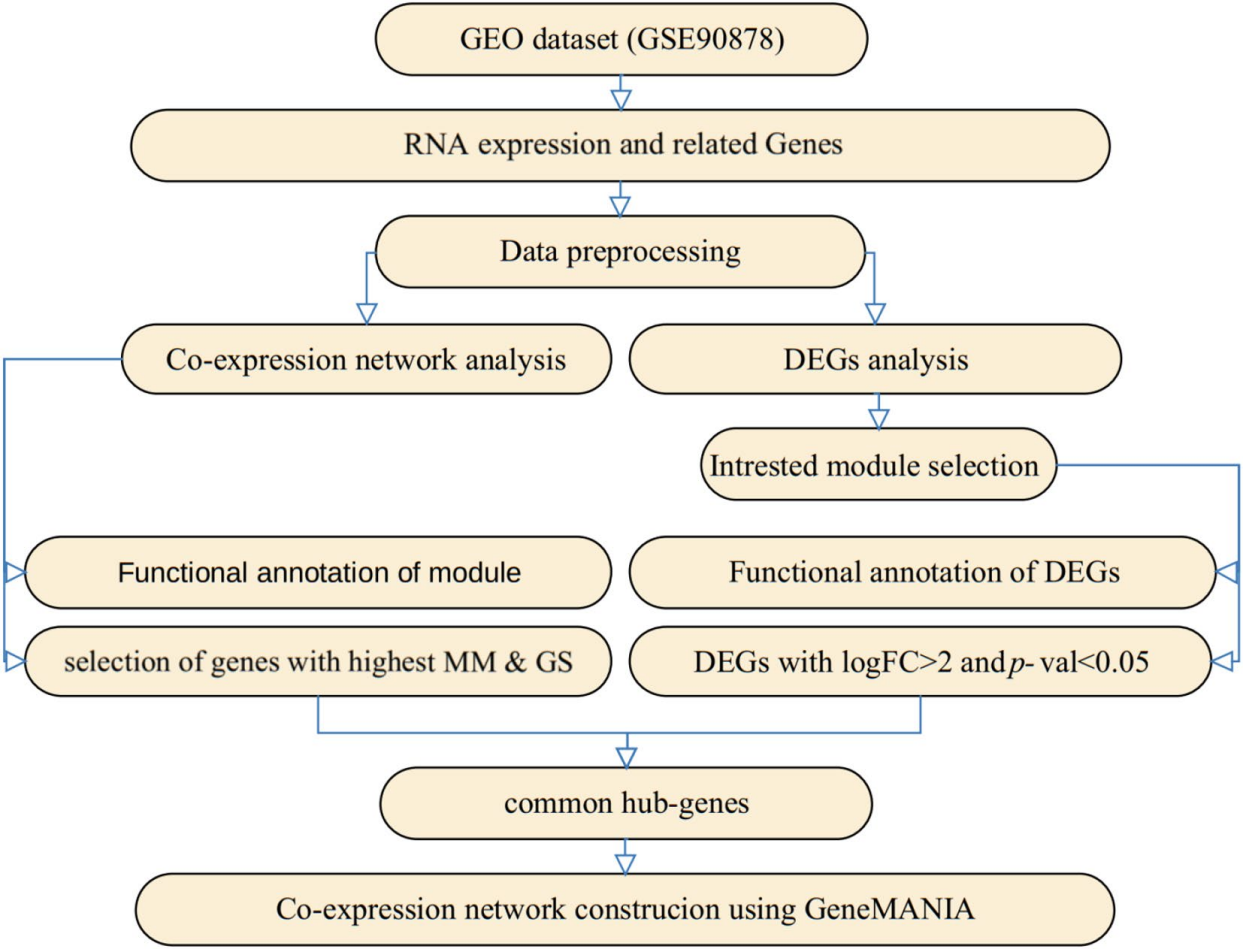
Flowchart showed preparation, processing, and interpretation of fetal and adult liver gene expression dataset and transcriptome in this study

The RNA-seq gene expression dataset GSE90878 was downloaded from the Gene Expression Omnibus (GEO) database (Lessard et al. 2017), generated using the Illumina HiSeq 2000 platform. This dataset contains raw data from 12 fetal liver erythroblast samples and 12 bone marrow erythroblast samples. Differentially expressed genes (DEGs) were identified using the DESeq2 package (version 1.18.1). Genes were considered DEGs if they met the following criteria: false discovery rate (FDR) < 0.001, and|log_2_ Fold Change| ≥ 2.5. The data analysis workflow is illustrated in Fig. 1.

Gene co-expression network of fetal liver erythroblasts and adult bone marrow erythroblasts were constructed as a control group using Weighted Gene Co-expression Network Analysis (WGCNA). Briefly, Pearson correlation coefficients were calculated for all pairs of genes. Subsequently, the gene expression profile matrix was transformed into a similarity matrix and then into an adjacency matrix. A minimum possible *p*-value was determined based on the stablished scale-free topology criterion. The topological overlap matrix (TOM) and dissimilarity TOM (diss TOM) were constructed using TOM similarity and dissimilarity measures for subsequent analysis. Module identification was performed using the dynamic tree cut algorithm with a minimum module size set to 30 genes. Modules exhibiting high similarity score were merged with threshold value of 0.2.

To identify modules significantly associated with the evaluated clinical features, we employed Module Eigengene (ME). MEs summarize the expression profiles of each module by representing the first principal component of the expression matrix. The association of individual genes with fetal erythroblast developmental stages to adulthood was assessed using gene significance (GS) values. Module Membership (MM) was defined as the correlation between the ME and the gene expression profile for each module. In this study, genes with the highest GS and MM were considered candidate hub-genes if they were also differentially expressed between fetal and adult samples. A Venny diagram was generated using the online tool Venny software (version 2.1; http://bioinfogp.cnb.csic.es/tools/venny/) to visualize the overlap between differentially expressed genes (DEGs) and hub genes. Functional networks were constructed using GeneMANIA (https://genemania.org/) to explore potential interactions between these genes. The resulting networks were visualized with Cytoscape software (version 3.0).

### Functional enrichment of significant module and preservation analysis

To identify and visualize enriched Gene Ontology (GO) terms, KEGG pathways, and biological pathways within the gene modules of interest, the ClueGO (version 2.2.5) plug-in was employed within the Cytoscape (version 3.6.0) environment. A kappa score of 0.4 was used as the threshold for significance. To mitigate potential biases arising from dataset-specific variations in gene expression, a module preservation analysis was conducted to validate the identified gene modules. The GSE109186 dataset was utilized as an independent validation set. The top 4,000 genes with the highest coefficient of variation were selected as input for the module preservation analysis. The degree of module preservation was quantified using Z_summary_ statistics. A Z_summary_ value less than 2 indicates no preservation, values between 2 and 10 suggest weak to moderate preservation, and values greater than 10 provide strong evidence of module preservation.

### Cell culture, treatment and viability determination

The human erythroleukemia cell line K562 (IBRC C10081) was obtained from the Iranian Biological Research Center. Cells were cultured in RPMI 1640 medium (Gibco, Invitrogen, US) supplemented with 10% (v/v) fetal bovine serum (Gibco; Invitrogen, US), 100 U mL^− 1^ penicillin, and 100 mg mL^− 1^ streptomycin (AriaCELL, Iran). Cultures were maintained at 37 °C in a humidified atmosphere with 5% CO_2_. For experimental treatment, 5 × 10^5^ cell mL^− 1^ were seeded in each well of a 6-well plate. Cells were treated with HU (Molecule, Germany) and Met (Merck-Germany) at concertation of 50, 100, and 150 µM for 24, 48, and 72 h. Each treatment condition was performed in replicate. Untreated cells served as controls.

The Cell viability and growth kinetics were assessed by determining the number of viable cells mL^− 1^ using a hemocytometer and the trypan blue dye exclusion method. An equal volume of cell suspension was mixed with a 0.4% trypan blue solution. Following a brief incubation, the mixture was loaded onto a hemocytometer, and viable (unstained) cells were counted under a light microscope.

### Apoptosis assay

K562 cells were treated with HU and a combination of HU/Met for 24 h. Following treatment, viable cells were harvested and subjected to Annexin V and propidium iodide (PI) double-staining. Annexin V-FITC emissions were detected on the FL-1 channel (530/30 nm), while PI emissions were detected on the FL-3 channel (585/40 nm). Approximately 10^4^ events were acquired in list mode on logarithmic scales for each sample using a Coulter Elite flow cytometer. Data analysis was performed to assess apoptosis and necrosis based on Annexin V and PI staining patterns.

### Erythroid differentiation assay

K562 cells were treated with HU for 24 h. Subsequently, cells were washed twice with phosphate-buffered saline (PBS) and resuspended in 10 mL PBS containing 0.5% bovine serum albumin (BSA). For flow cytometry analysis, 2 × 10^5^ cells were incubated with CD71-FITC and CD235a-PE antibodies (Affymetrix, USA) for 30 min on ice. Following incubation, cells were washed twice with PBS containing 0.5% BSA. Unstained cells served as isotype control. Flow cytometry data were acquired on a flow cytometer and analyzed using the FlowJo 10.8.1 software suite.

### Hb F assay

Flow cytometry was employed to quantify the percentage of Hb F-positive cells (F-cells). On the 1st day of treatment, 2 × 10^5^ treated and untreated cells were collected. Cells were washed with PBS containing 0.5% BSA and subsequently fixed for 10 min in freshly papered 0.05% glutaraldehyde. Permeabilized was achieved by incubating the cells in 0.1% Triton X-100 for 3 min. Cells were then incubated with 5 µl of fluorescein isothiocyanate (FITC)-conjugated anti-Hb F antibody for 15 min at room temperature. Following two washes with PBS, stained cells analyzed on a flow cytometer (BD Biosciences, USA) in the FL1 channel.

### Detection of *GCNT2*, *IGF2BP1*, *γ-globin* genes expression and MiRNA by qRT-PCR

K562 cells were harvested, including both mRNA and miRNA, was extracted using the RNA and miR Extraction Kit according to the manufacturer’s instructions (GeneAll, South Korea). RNA quantity and quantity were assessed using spectrophotometry (Nanodrop, EPOCH, USA). Complementary DNA (cDNA) was synthesized from 1 µg of total RNA using oligo (dT) primers and M-MLV reverse transcriptase (Pars tous Biotechnology, Iran) according to the manufacturer’s protocols. Quantitative real-time PCR (qRT-PCR) was performed on a StepOne™ Real-Time PCR System (Applied Biosystems, Foster City, CA, USA), using SYBR green-based detection. To determine the relative gene expression levels of *GCNT2*, *IGF2BP1*, and *γ-globin*, as well as miR level, cDNA samples were mixed with the Ampliqon SYBR Green master mix (Ampliqon, Odense, Denmark) and specific Primers (Table 1). Amplification was performed in triplicates using the cycling conditions outlined in Table 2. The 2^-ΔΔCT^ methods was employed to calculated relative mRNA and miR expression levels, normalized to U6 RNA small nuclear RNA (U6 snRNA) expression.

**Table 1:** Primer sequence of GCNT2, IGF2BP1, γ-globin, and GAPDH genes used in qRT-PCR

Primer name	Sequence
GCNT2-F	5´- GTG AAA CTG CGA TAC AAC CC- 3´
GCNT2-R	5´- CCT GAA GCT ATA AAC GTG GTC- 3´
IGF2BP1-F	5´- CCT CCA TCA AGA TTG CAC CAC- 3´
IGF2BP1-R	5´- CCG TCA AAT TCT GCA ACT CGT- 3´
GAPDH-F	5´- GTC TCC TCT GAC TTC AAC AGC G- 3´
GAPDH-R	5´- ACC ACC CTG TTG CTG TAG CCA A- 3´
Hb F-F	5´- TTG GCA ACC TGT CCT CTG CCT- 3´
Hb F-R	5´- GAA ATG GAT TGC CAA AAC GGT CAC- 3´
miR144-3p-F	5’-CCC TAC AGT ATA GAT GAT GTA-3’
miR199-5p-F	5′-GTG CTC GCT TCG GCA GCA CAT-3′
miR-451-5p-F	5′-AAA CCG TTA CCA TTA CTG AGT T-3′
U6-snRNA-F	5’-CAA ATT CGT GAA GCG TTC CAT AT-3’

**Table 2:** qRT-PCR implementation program designed based on the sequence of gene primers

Gene	Step 1	Step 2 (40 cycles)	Step 3	Step 4
GCNT2	95 °C–5 min	95 °C–30 s 56 °C–30 s 72 °C–30 s	95 °C–5 min	60 °C–1 min
IGF2BP1	95 °C–5 min	95 °C–30 s 53 °C–30 s 72 °C–30 s	95 °C–5 min	60 °C–1 min
Hb F	95 °C–5 min	95 °C–30 s 59 °C–30 s 72 °C–30 s	95 °C–5 min	60 °C–1 min
miRs	95 °C–5 min	95 °C–30 s 60 °C–30 s 72 °C–30 s	95 °C–5 min	60 °C–1 min

### Statistical analysis

All statistical analyses were performed using GraphPad Prism 9 software (GraphPad Software, USA). Statistical significance was determined using one-way analysis of variance (ANOVA), with a significance level of *P* < 0.05. Data presented as the mean ± standard error of the means (SEM).

## Results

### Preprocessing and DEGs analysis

**Fig. 2:**
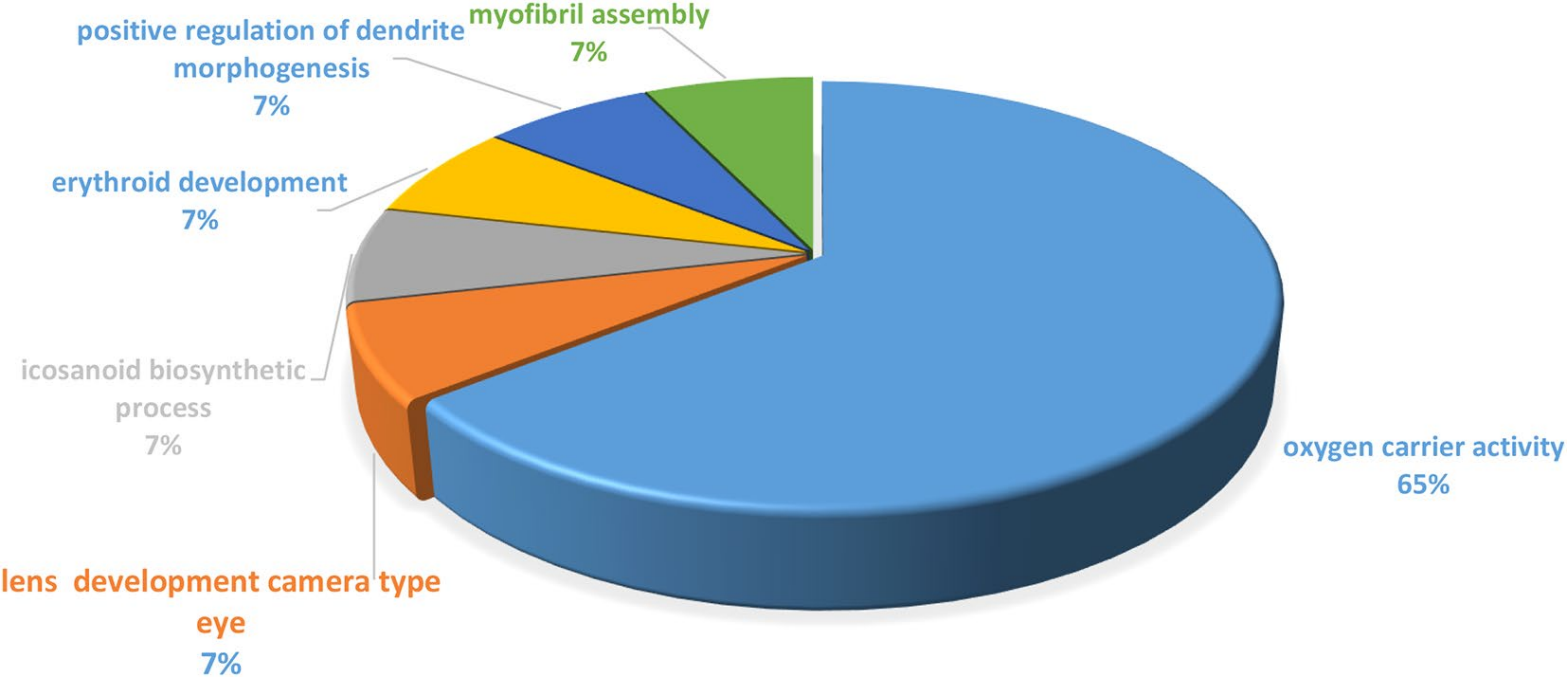
Identification of the biological pathway and KEGG analysis of DEGs based on the 65% of genes involved in the oxygen carrier pathway

To minimize the impact of variability, we performed quantile normalization on the gene expression data. Sample clustering revealed no outliers; therefore, all samples were included in subsequences analysis. DEG analysis between fetal liver and bone marrow erythroblast identified 93 DEGs (65 upregulated, 28 downregulated). These DEGs were selected for further investigation. Gene Ontology (GO) and Kyoto Encyclopedia of Genes and Genomes (KEGG) pathway enrichment analyses were conducted to elucidate the biological and pathways associated with the DEGs (Fig. 2). GO analysis categorizes The DEGs into three main domains: biological processes, molecular functions, and cellular components, providing insights into their functional roles within specific cellular contexts. KEGG pathway analysis mapped the DEGs onto known metabolic and signaling pathways, revealing the underlying biological mechanisms driving the observed gene expression changes. Collectively, these analyses provide a comprehensive understanding of the molecular and cellular processes involved in the differential gene expression between fetal liver and bone marrow erythroblasts.

### Identification of WGCNA modules

**Fig. 3:**
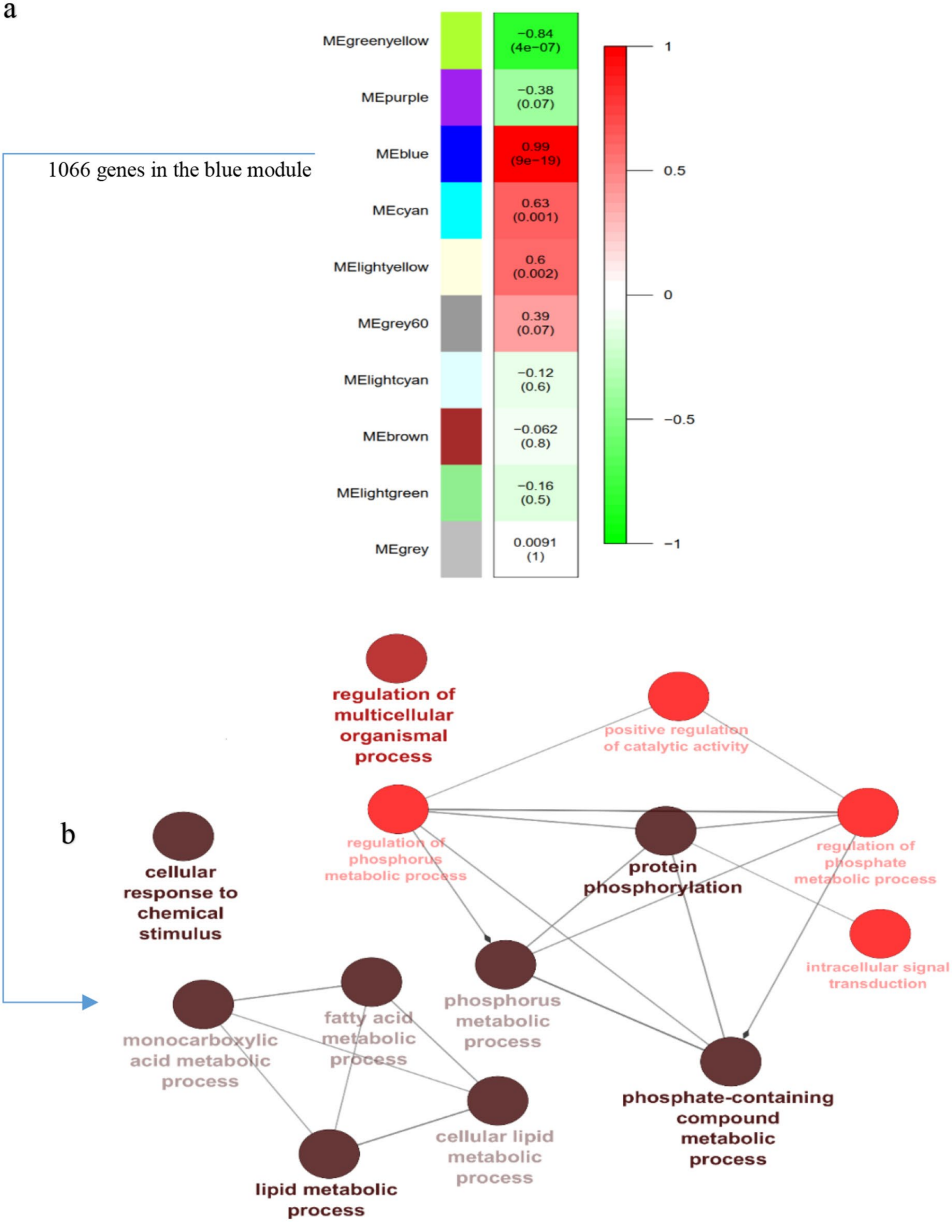
Module-trait relationships and enrichment. **(a)** Each row represents a module eigengene and each column represents fetal globin switching. The numbers in each cell indicate the correlation and p-value; **(b)** The blue color represents the biological processes and pathways detected. The size of the node indicates the number of genes in each pathway, and the color of the node indicates statistical significance. A darker pathway node indicates greater statistical significance, with a gradient from red (p-value 0.05 − 0.005) to black (p-value < 0.0005)

Based on the variance of expression values, 4,000 genes were selected for WGCNA. A soft-threshold power of 7 (β = 7) was chosen to construct a scale-free network, and the weighted gene co-expression network of fetal liver and adult erythroblast samples were subsequently reconstructed. Using the dynamic tree cut algorithm, 10 co-expressed gene modules were identified by hierarchical clustering (Fig. 3a). GO and KEGG pathway enrichment analysis were performed using ClueGO to investigate the biological relevance of the blue module. Functional enrichment analysis revealed significant associations with biological processes such as haptoglobin binding and erythroid development (Fig. 3b). In addition, pathways involving Protein phosphorylation is a key regulatory mechanism in cellular signaling pathways, including those involved in erythropoiesis and erythroid cell differentiation. Phosphorylation modulates the activity of key regulatory proteins, influencing erythroblast Developmental stages and hemoglobin production. This, in turn, impacts haptoglobin binding, a crucial process for the clearance of free hemoglobin and the maintenance of iron homeostasis during erythrocyte turnover.

**Fig. 4:**
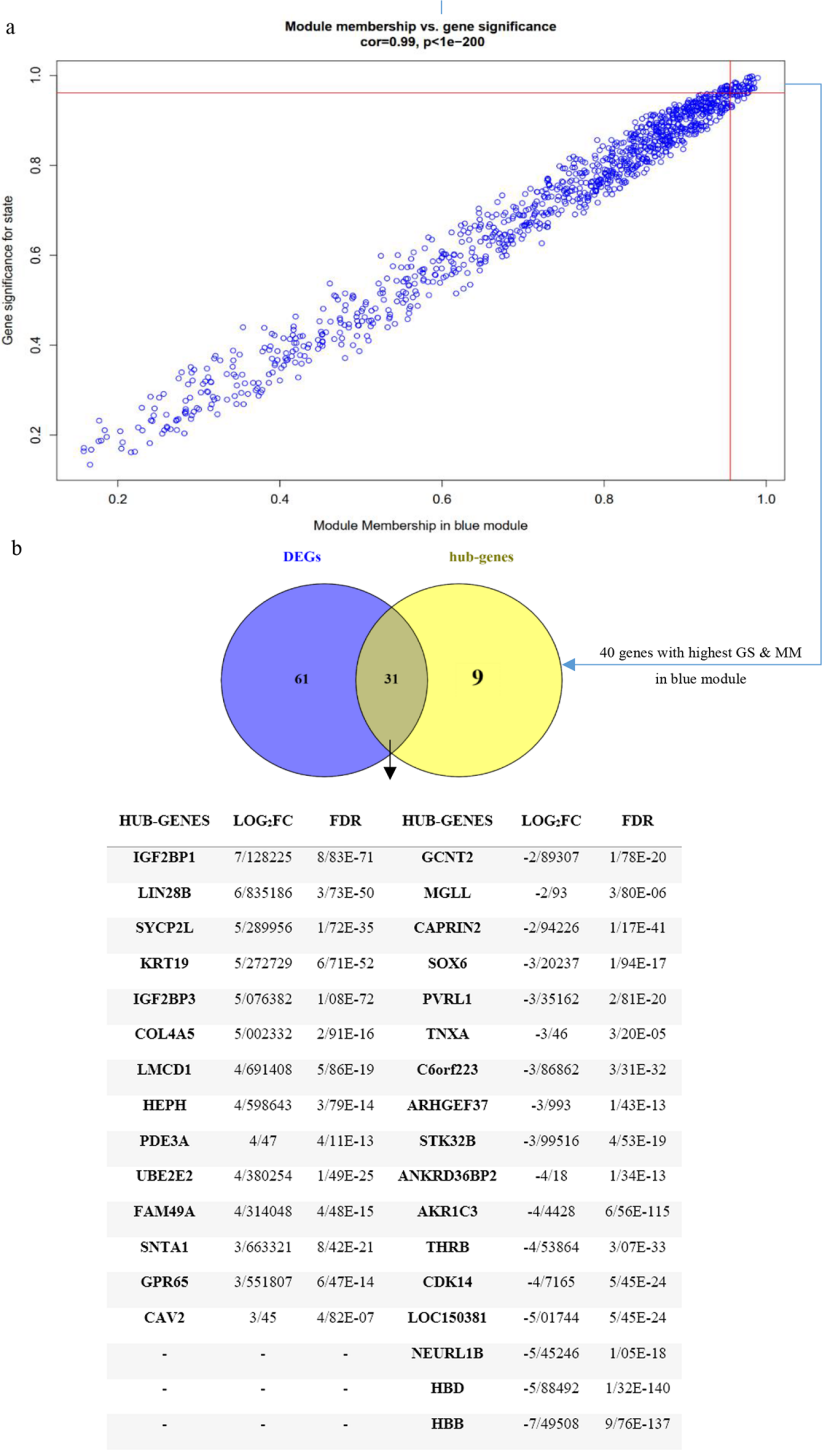
Hub genes detection. **(a)** Blue module features of G.S and M.M which significantly correlated with erythroid Development. Each point represents an individual gene within each module, which is plotted by G.S on the y-axis and MM on the x-axis; (**b)** Evaluation of similarity between DEGs and Hub genes lists using a Venn diagram. Thirty-one genes that were similar in both lists were then imported to Gene MANIA to construct a co-expression network

Module sizes ranged from 18 (grey) to 1,218 (green, yellow) (Table 3). The blue module exhibited the strongest positive correlation with erythroblast development (*r* = 0.99, *p* = 1.00 e-200) (Fig. 4a).

**Table 3:** Module colors characterization. Displaying the information including; the number of genes, correlation, and p-value in different modules

Module colors	Number of genes	Correlation	*p*-value	Z_summary_
Blue	1060	0.99	9E-19	12
Brown	855	0.062	0.8	-0.21
Cyan	104	0.63	0.001	0.22
Green yellow	1218	-0.83	4E-07	10
Grey	18	0.0001	1	8.80
Grey60	192	0.39	0.07	3.10
Light cyan	84	-0.12	0.6	0.86
Light green	177	-0.16	0.5	1.70
Light yellow	171	0.6	0.002	-0.54
Purple	45	-0.38	0.07	-0.61

Lipid metabolic processes, particularly those involved in membrane synthesis and signaling, are crucial for proper erythroid cell maturation. Lipid metabolism has been implicated in hematopoietic stem cell differentiation, including commitment to the erythroid lineage. Alterations in lipid metabolism can significantly influence erythroblast differentiation and maturation, linking these pathways to the development of erythrocytes and associated functions such as hemoglobin binding. These results suggest that the blue module plays a critical role in regulating erythroid development. Protein phosphorylation and lipid metabolism emerge as key contributors to this process, alongside haptoglobin binding.

### Hub gene identification and network analysis of interesting modules

**Fig. 5:**
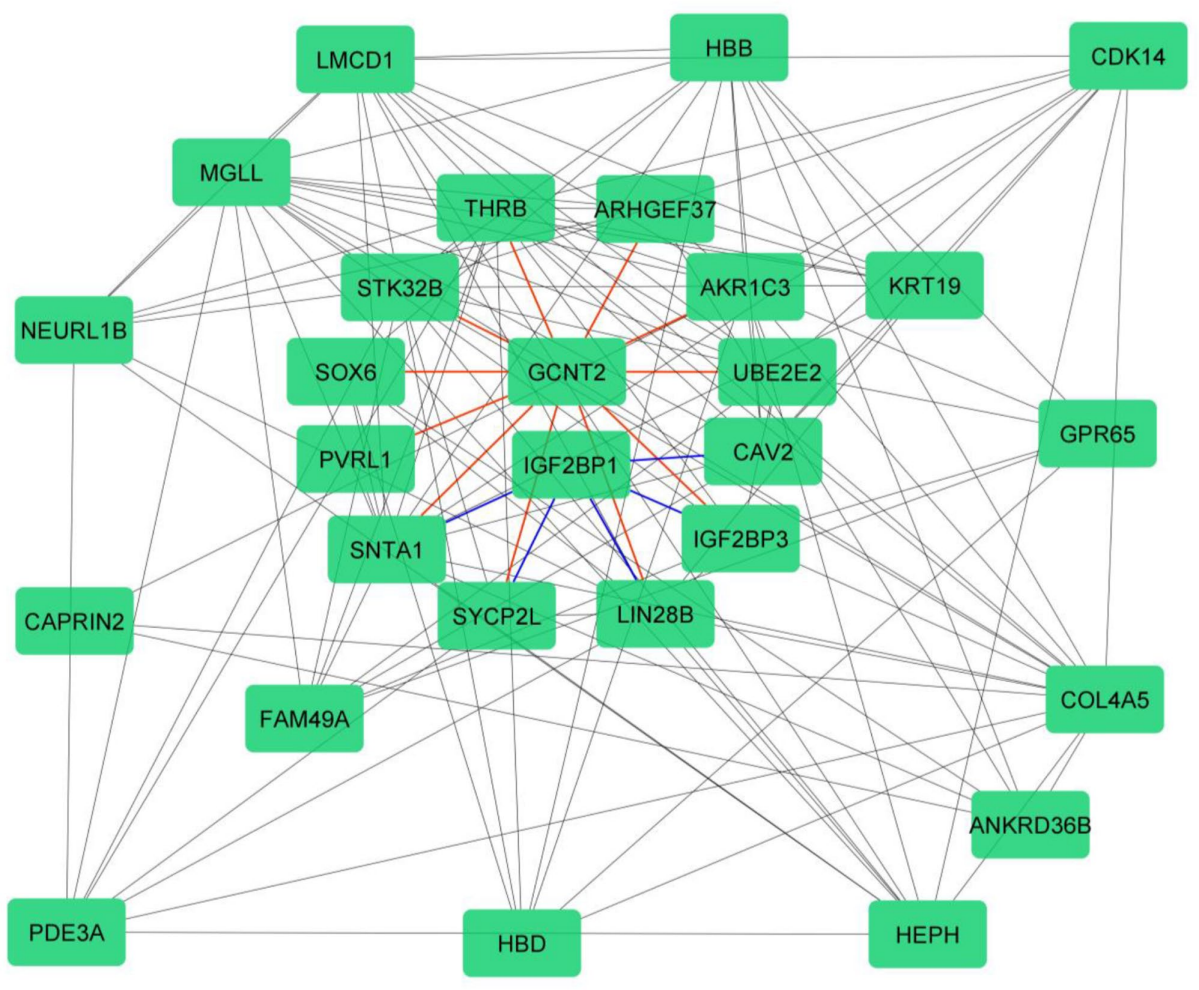
Visualize interactions between the hub-genes in a co-expression blue module using GeneMAINA. The gene relationships indicate that IGF2BP1 and GCNT2 genes play a significant role in the γ-globin switching pathway by influencing the synthesis of the gamma chain. These genes are among the important genes involved in this pathway

The correlation between the M.M and G.S scores of the selected modules (Fig. 4a) identified genes positively associated with erythroid Development. Forty-one genes with the highest M.M and G.S scores within the blue module were then compared to the DEGs list. Genes identified in both lists were considered as final hub genes (Fig. 4b). These hub genes were then imported into the GeneMANIA database to visualize co-expressed network of gene module. The resulting gene co-expression network is depicted in Fig. 5. These findings suggest that the identified relevant genes are genetically interconnected. Furthermore, the selected target genes exhibit numerous genetic associations with one another, indicating the formation of a significant gene network.

### Module preservation analysis

Module preservation analysis using the GSE98787 dataset revealed a Z_summary_ score of 12 for the blue module (Table 3 and Figure S5). This high Z_summary_ score indicates that the blue module, significantly associated with γ-globin switching in our study, exhibits strong preservation in an independent dataset, suggesting its biological relevance and robustness.

### HU induces K562 erythroid differentiation

**Fig. 6:**
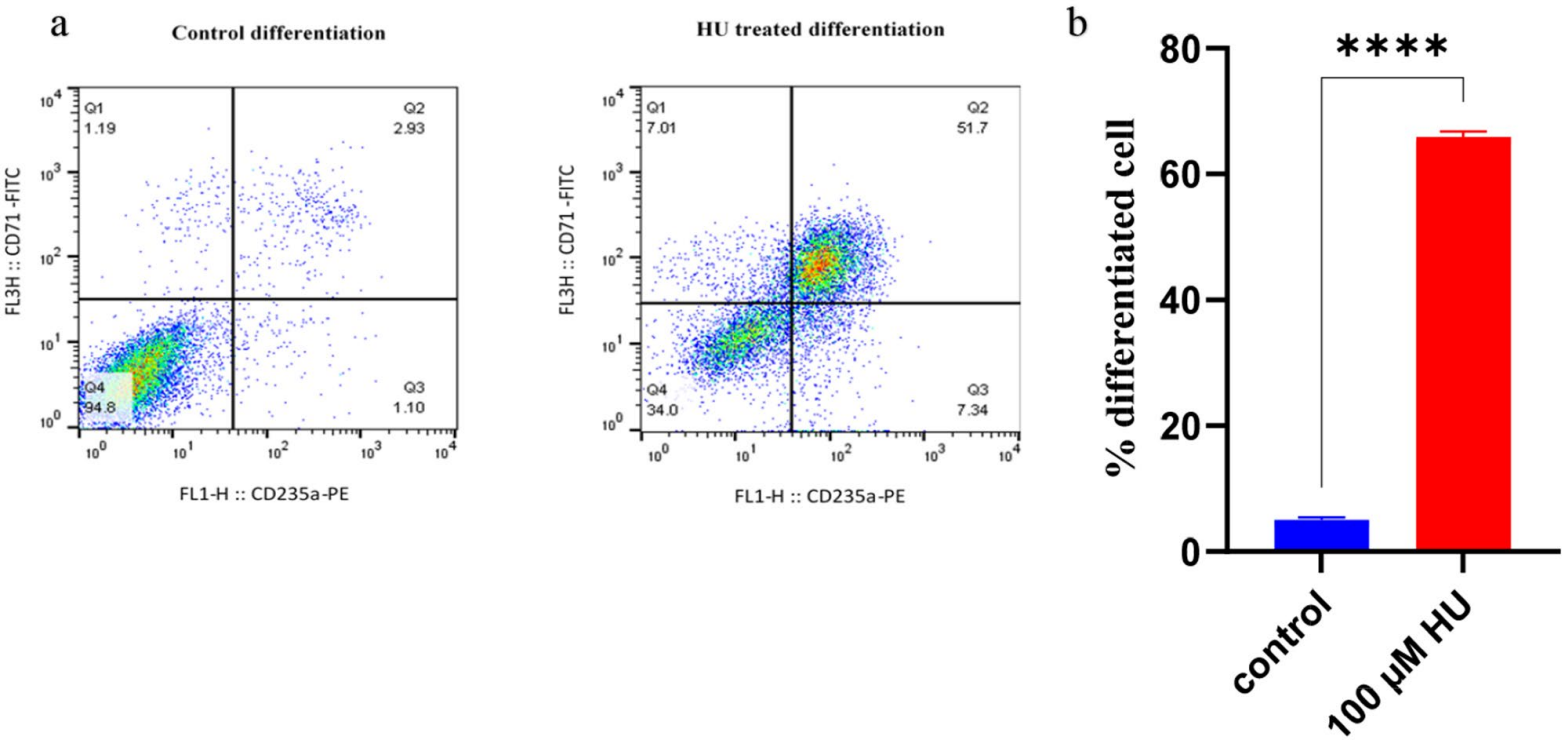
K562 cell differentiation under HU-treatment. **(a)** cell differentiation assay by CD235 and CD71 in HU treated cells. **(b)** differentiated cells percentage compare with the control group. HU (100 µM) treated cells were going to differentiation 66% compared to 5.2% in the control group. (**** *p*-value < 0.0001)

To investigate the impact of HU on the process of erythroid differentiation in K562 cells, the expression levels of CD71 and CD235a were assessed using flow cytometry. Treated with 100 µM HU significantly enhanced erythroid differentiation. As shown in Fig. 6, HU treatment resulted in a notable increase in the proportion of CD235a^+^ and CD71^+^ cells, reaching 66.7% compared to the control group. As shown in Fig. 6, HU treatment resulted in a notable increase in the proportion of CD235a^+^ and CD71^+^ cells, reaching 66.7% compared to the control group.

### Effect of HU on K562 cells growth and viability

**Fig. 7:**
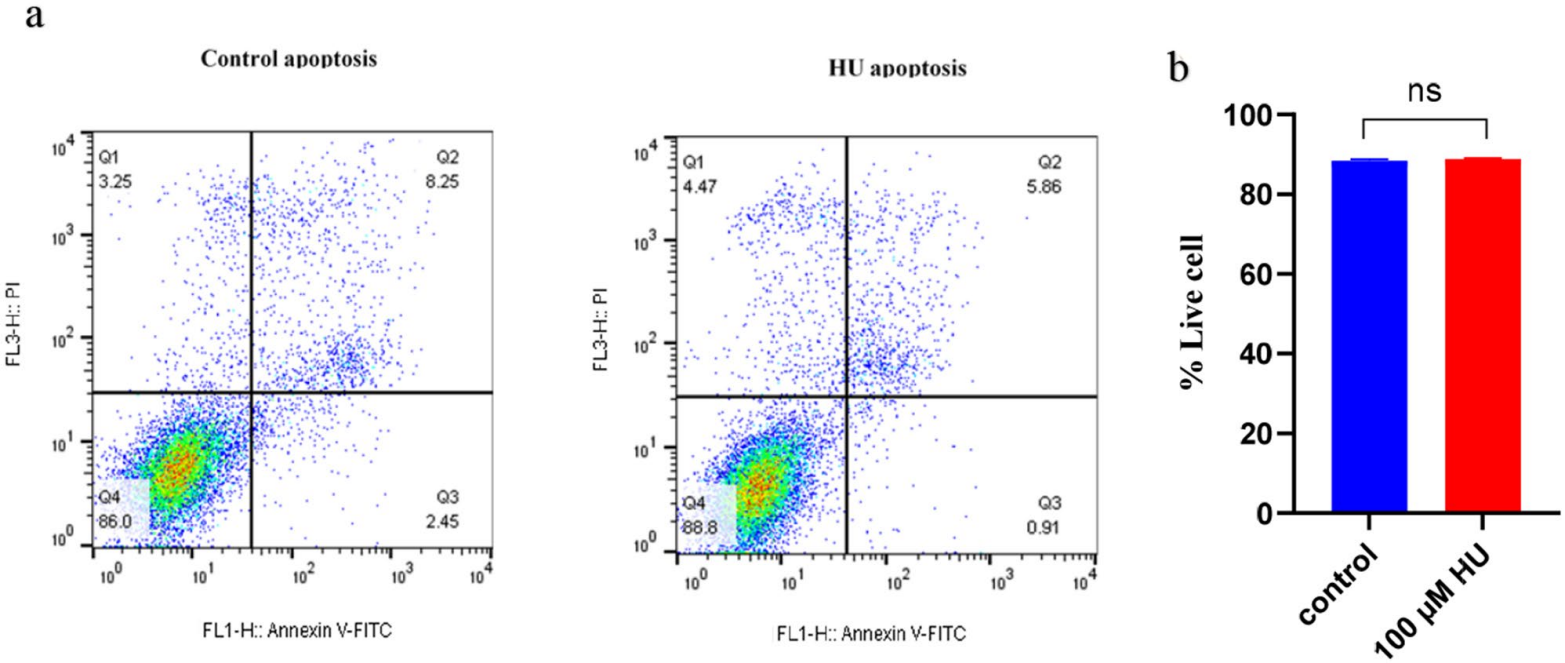
K562 cells viability under HU treatment. **(a)** cell viability assayed by Annexin V and PI using flow cytometry in HU treated and control groups. **b)** the viability in HU-treatment group compared to the untreated group no significant difference observed (*p*-value > 0.05)

To assess the cytotoxic effects of HU on K562 cells, cells were treated with 100 µM HU. Cell viability was subsequently evaluated using flow cytometry with Annexin V and PI staining. The results demonstrated no significant alterations in cell viability in the treated groups compared to the untreated control (Figs. 7a, b).

### HU and Met induce γ-globin transcription and hb F production in K562 cells

**Fig. 8:**
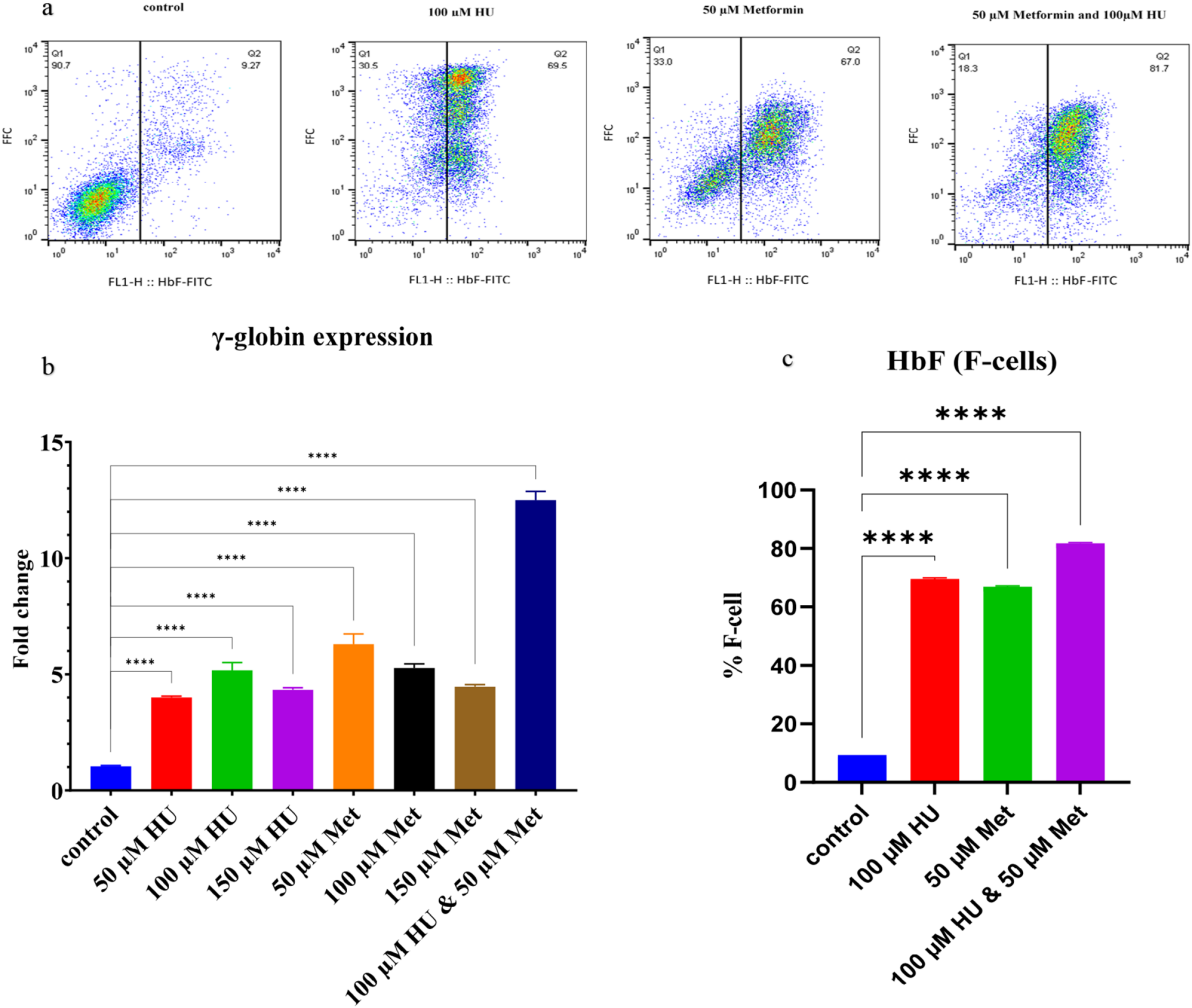
Hb F percent and γ-globin expression in HU, Met and HU/Met treated cells. **(a)** Hb F assay using flowcytometery, the F-cell percent in the HU-treated cells (69.5%), Met (67.0%) and HU/Met (81.7%) compare to control group (9.2%). **(b)** γ-globin gene expression by qRT-PCR in treated cells. **(c)** the percent of F-cells in the HU, Met and HU/Met treated cells vs. control group (**** *p*-value < 0.0001)

Treatment with 100 µM HU, 50 µM Met, and their combination for 24 hours significantly increased γ-globin expression by 5.16, 6.23, and 11.5-fold, respectively, as shown in Fig. 8b. furthermore, immunophenotyping analysis revealed a substantial increase in the F-cell population by 53.07%, 61.3%, and 81% following treatment with 100 µM HU, 50 µM Met, and the combination, respectively, as depicted in Fig. 8a and c.”

### GCNT2 and IGF2BP1 can modulate the γ-globin and contributes to γ-globin elevation in K562 cells

**Fig. 9:**
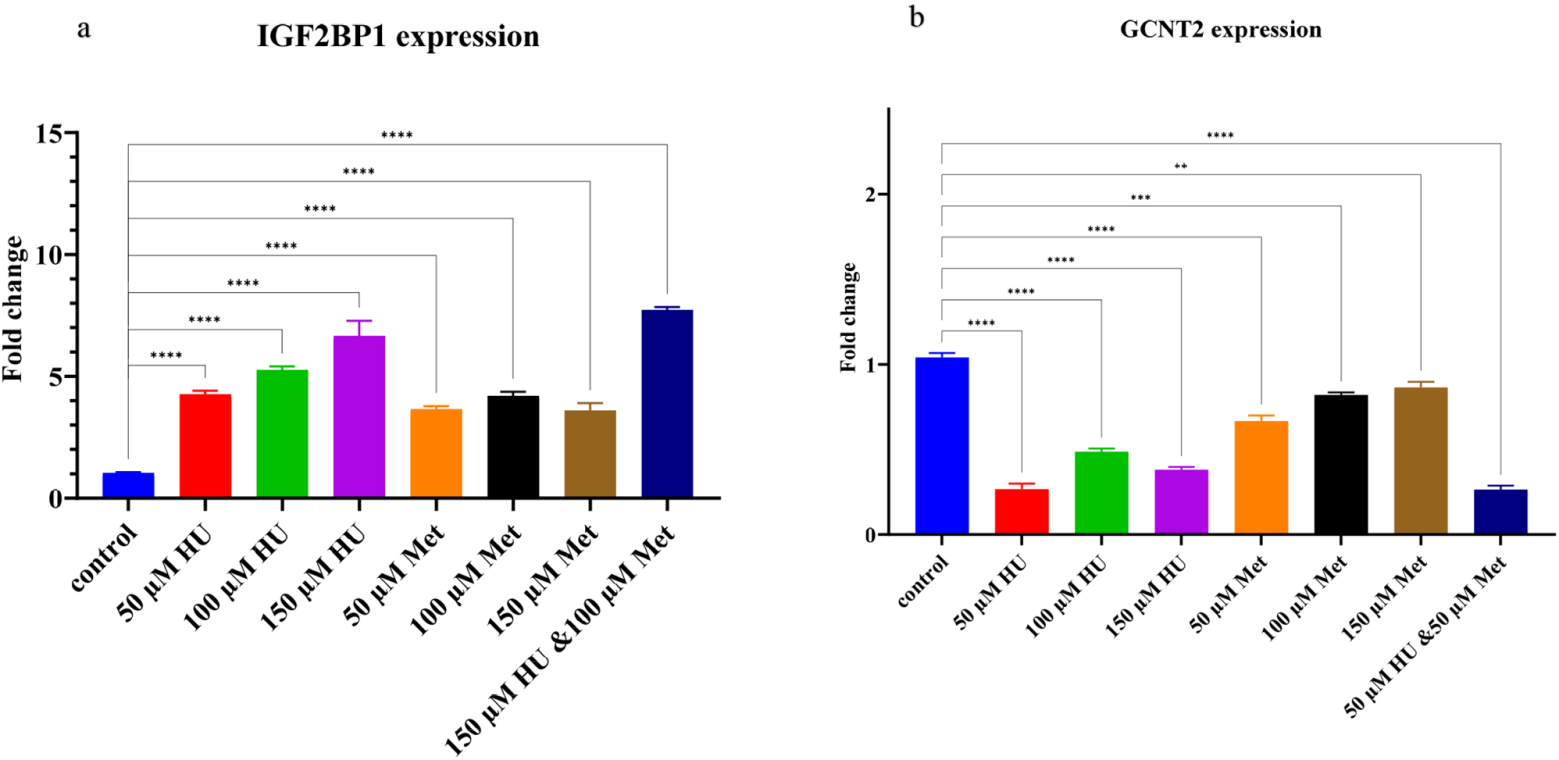
IGF2BP1 and GCNT2 expression by qRT-PCR. **(a)** IGF2BP1 expression level in the HU, Met and HU/Met treatment cells compared to the untrated cells. **(b)** GCNT2 gene expression in the HU, Met and HU/Met treated cell compare to the control group. (** *p*-value < 0.01, *** *p*-value < 0.001, **** *p*-value < 0.0001)

The expression levels of the *IGF2BP1* and *GCNT2* genes were determined in K562 cells treated with HU and Met. Treatment with 150 µM HU, 50 µM Met, and their combination (150 µM HU/50 µM Met) resulted in a 6.2, 4.3, and 7.3-fold increase in *IGF2BP1* expression, respectively, after 24 h. In contrast, *GCNT2* expression decreased by 0.3, 0.7 and 0.26-fold, respectively, in the presence of 50 µM HU, 50 µM Met and the HU/Met combination (50 µM/50 µM) compared to the control group (Figures. 9a, b).

### miR-199a/b-5p, miR-451-5p and miR-144-3p contributes to γ-globin elevation in K562 cells

**Fig. 10:**
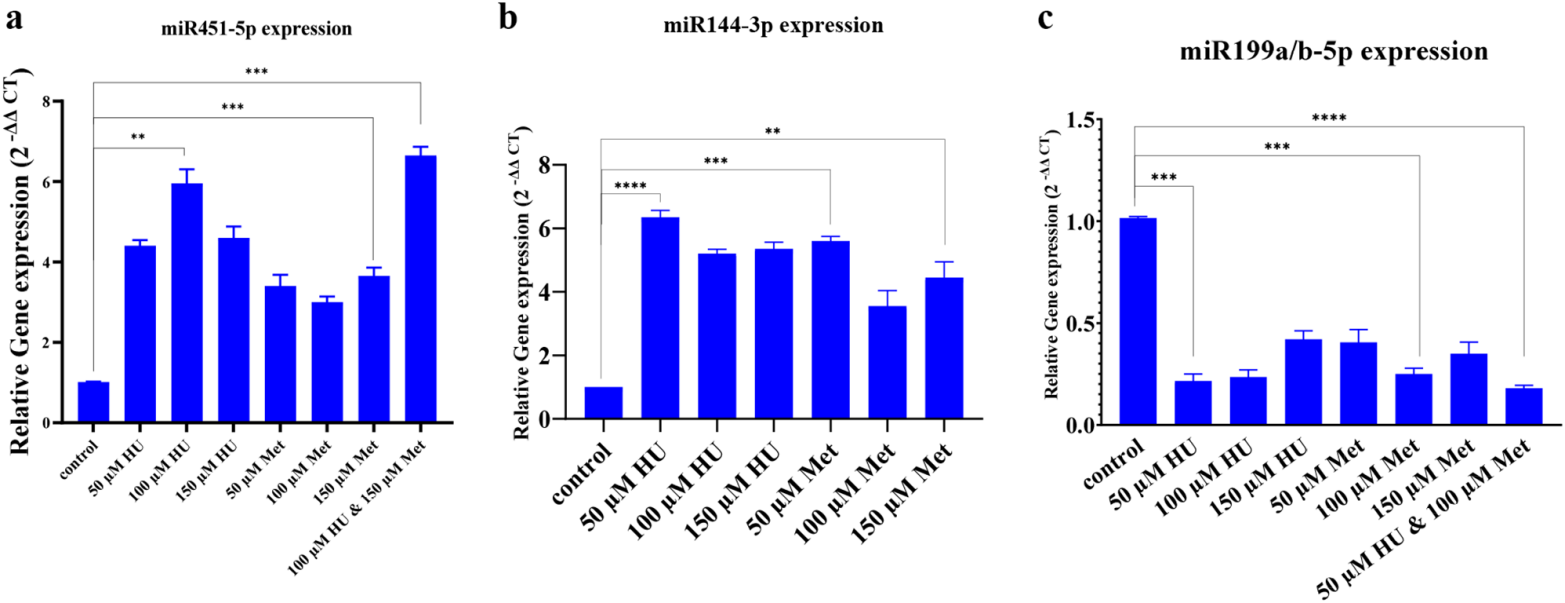
miR199a/b-5p, miR451-5p and miR 144-3p expression. The miR451-5p **(a)**, miR144-3p **(b)**, and miR199a/b-5p **(c)** expression level in the HU, Met and HU/Met treated cell. (**p-value < 0.01, ***p-value < 0.001, ****p < 0.0001)

The results revealed a significant upregulation of miR-451-5p and miR-144-3p in k561 cells treated with HU/Met (100 µM/50 µM) and HU (50 µM), respectively. In contrast, miR-199a/b-5p expression was significantly downregulated in cells treated with HU/Met (100 µM/50 µM) compared to the control group (Figures. 10a, b, c).

## Discussion

Reactivating HbF expression through small molecule compounds offers a promising therapeutic approach for hemoglobinopathies. Compounds such as 5-azacytidine, short chain fatty acid derivatives, arginine butyrate, and decitabine have demonstrated Hb F inducing activity in clinical trials. These agents exert their effects through various mechanisms, including histone deacetylase (HDAC) inhibition and DNA methyltransferase (DNMT) inhibition, activation of cell signaling pathways, and modulation of transcription factors binding DNA. The development of novel, effective, and safe oral medications capable of inducing Hb F alone or in combination with HU hold significant potential for improved outcomes in hemoglobinopathies. The K562 cell line serves as an in vitro model system to identify novel Hb F inducers and investigate their underlying molecular mechanisms.

Initially, the CD235a-CD71 assay was employed to evaluate erythroid differentiation in K562 cells. The results demonstrated that treatment with HU induced erythroid differentiation in K562 cells, as evidenced by a significant increase in the proportion of CD235a^+^ cells. Importantly, this differentiation occurred without affecting cell viability, proliferation, or growth kinetics. Our results further indicated that erythroid differentiation was associated with increased *γ-globin* mRNA synthesis, and higher levels of Hb F were correlated with enhanced γ-globin synthesis.

Previous studies have demonstrated that *IGF2BP1*, an RNA-binding protein, downregulates *BCL11A*, a critical inhibitor of HbF expression (Rini and Anbalagan 2017). *IGF2BP1* exerts its function through various mechanisms. It interacts with target mRNAs, including actin, cytoplasmic 1, c-MYC, CD44, and MKI67, thereby influencing cellular processes such as proliferation, differentiation, signaling, and migration. While *IGF2BP1* downregulation of BCL11A was initially thought to involve post-translational modifications, subsequent studies have shown that IGF2BP1 is sequestered within cytosolic granules containing essential translation factors, such as eIF4 and the 60 S ribosomal subunit, leading to translational inhibition of BCL11A. Furthermore, IGF2BP1 can modulate chromatin architecture by interacting with key transcription factors like LDB1, GATA1, and FOG1, facilitating the repositioning of the Locus Control Region (LCR) from the beta to the gamma-globin promoter and enhancing gamma-globin gene expression. During human evolution, a shift in the LCR junction towards the pre-beta region, likely associated with reduced *IGF2BP1* expression, contributed to decreased HbF expression and increased adult hemoglobin (HbA) expression. Independent studies have implicated other factors in HbF regulation. de Vasconcellos et al. demonstrated an inverse correlation between *GCNT2* expression and Hb F levels, suggesting a role for this glycosyltransferase in HbF repression (de Vasconcellos et al. 2017). Similarly, Y. Terry Lee et al. observed that induction of *LIN28B* expression, which inhibits let-7 microRNA function, is associated with decreased expression of *GCNT2* and *CA1* genes and concomitant increases in HbF (Lee et al. 2013). Met, has been shown to induce HbF expression in erythroid precursors. This effect appears to be mediated through the FOXO3 pathway, as Met treatment does not significantly alter the expression levels of key erythroid transcription factors such as BCL11A, MYB, or KLF1.

Due to the short 3′ UTRs of globin genes, our understanding of miRNA-mediated regulation of globin gene expression remains limited. While miR-96 directly silences γ-globin gene expression by binding to its coding sequence (CDS), few miRNAs have been identified that target globin loci. Moreover, Moreover, some studies have demonstrated indirect regulation of globin genes by miRNAs through the targeting of key transcription factors. For instance, miR-144 negatively regulates embryonic β-globin expression by targeting KLF1, while miR-15a and miR-16-1 enhance HbF expression in human trisomy through the downregulation of MYB (Ma et al. 2013). This study investigated the expression levels of miR-451-5p, miR-144-3p, and miR-199a/b-5p in K562 cells treated with HU and Met. Our findings revealed a significant upregulation of miR-451-5p and miR-144-3p, while miR-199a/b-5p expression was significantly downregulated. These observations suggest that these miRNAs may play crucial roles in regulating the expression of key transcription factors involved in the γ-globin gene regulatory network.

This study utilized a preclinical model employing HU and Met to evaluate their potential to enhance γ-globin mRNA levels and HbF production. The effects of these agents on erythroid differentiation, proliferation, γ-globin gene expression, and HbF synthesis were investigated in the K562 cell line. Under standard cell culture conditions, the K562 cell line exhibits low levels of hemoglobin synthesis. However, exposure to inducing agents can trigger an increase in erythroid differentiation characteristics. The incubation of K562 cells with HU and Met resulted in a significant increase in γ-globin expression and HbF production.

**Fig. 11:**
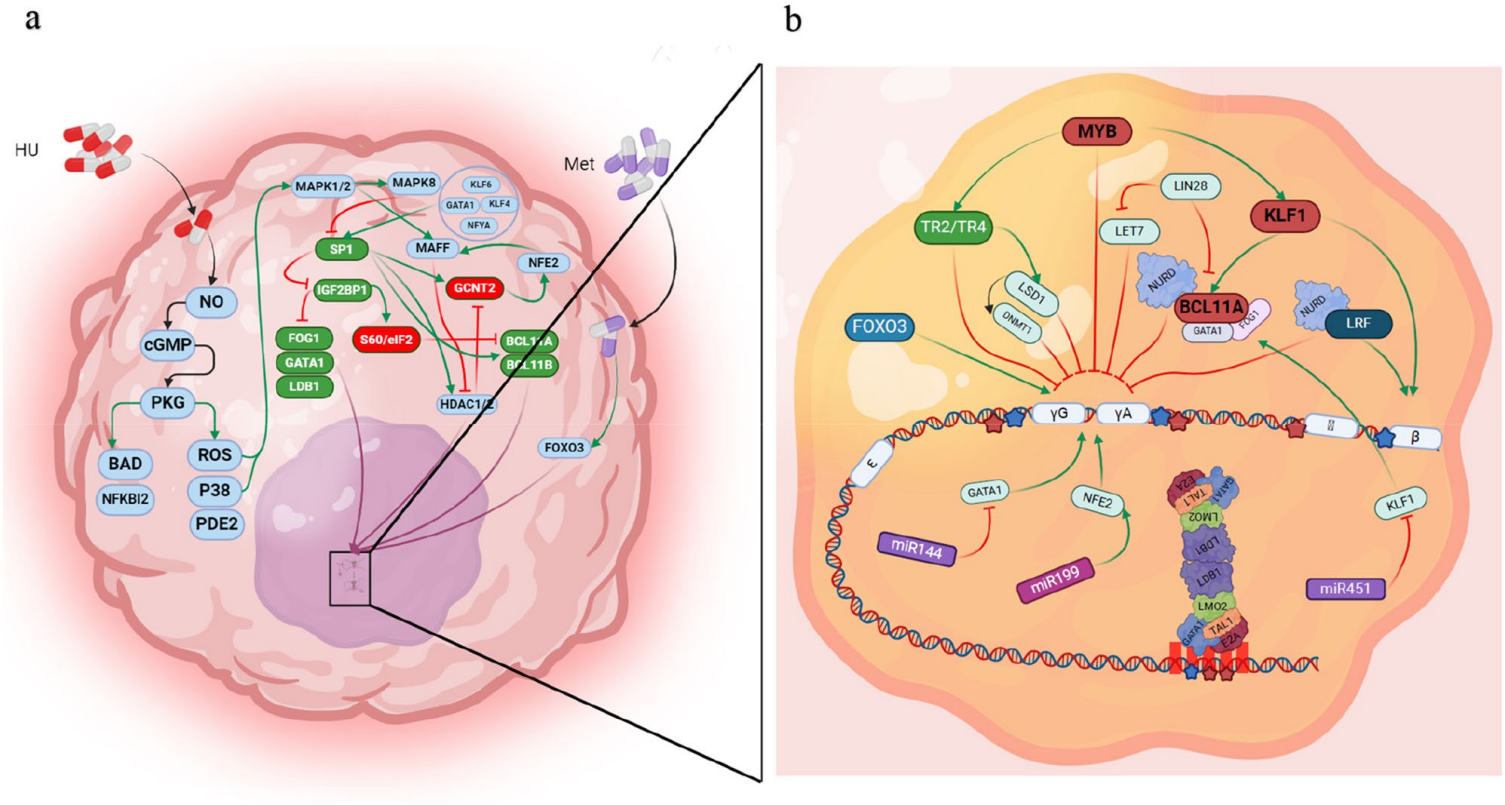
The impact of hydroxyurea and metformin on γ-globin induction involves complex molecular pathways. (**a**) Upon entering the cell, hydroxyurea enhances the production of ROS and activates P38 through modulation of the PKG pathway, subsequently leading to activation of the RAS-MAPK signaling pathway. This ultimately results in the downregulation of SP1, which in turn increases IGF2BP1 and decreases GCNT2. Consequently, this signaling cascade inhibit the activity of HDAC and DNMT. On the other hand, metformin increases the expression of FOXO3. (**b**) Additionally, KLF1, activated by BCL11A, displaces the LCR from the beta gene, leading to the suppression of gamma gene expression and a decrease in hemoglobin F production. LIN28 inhibits BCL11A and promotes gamma gene expression. The combined effect of hydroxyurea, including the activation of HDAC and DNMT1, and the inhibition of BCL11A, leads to chromatin remodeling and increased gamma gene expression

This study demonstrated that HU and Met significantly increased γ-globin gene expression and HbF production within the first 24 h of treatment. While the precise molecular mechanisms underlying this effect remain to be fully elucidated, our findings suggest that HU and Met modulate the expression of key regulatory genes, including *GCNT2* and *IGF2BP1*, leading to enhanced HbF levels (Khandros et al. 2020). HU treatment was associated with increased levels of ROS and activation of the MAPK8 pathway, while Met treatment activated the AKT kinase pathway, which is known to indirectly inhibit the activity of the SP1 transcription factor (Cokic et al. 2003). n silico analyses indicated potential binding sites for SP1 within the promoter regions of *IGF2BP1* and *FOXO3* genes, suggesting that HU and Met may downregulate *SP1* expression. Previous studies have established that IGF2BP1 functions as an inhibitor of GCNT2. Our findings support this observation, demonstrating that increased *IGF2BP1* levels in HU-treated cells are associated with decreased *GCNT2* gene expression. Furthermore, *IGF2BP1* enhances *γ-globin* expression by modulating the translation of key cellular proteins, including BCL11A, and influencing the spatial relationship between the LCR and the γ-globin gene (Chambers et al. 2020). Therefore, we propose that the observed reduction in cell glycosylation, potentially linked to decreased *GCNT2* expression, and the modulation of key regulatory factors, including IGF2BP1, may contribute to the observed enhancement of γ-globin expression in the absence of GCNT2 (Fig. 11).

While this study demonstrates the potential of HU and Met to enhance γ-globin mRNA levels and HbF production in K562 cells, it is essential to acknowledge inherent limitations associated with the employed vitro model. The K562 cell line, derived from chronic myelogenous leukemia, exhibits an aberrant genetic profile, potentially influencing its response to differentiating agents. Consequently, direct extrapolation of our findings regarding HU and Met’s effects on erythroid differentiation and γ-globin induction to primary human erythroid cells or in vivo systems warrants careful consideration. Furthermore, although significant alterations in the expression of miR-451-5p, miR-144-3p, and miR-199a/b-5p were observed following HU and Met treatment, the precise downstream targets and functional implications of these miRNA modulations on γ-globin regulation remain to be determined. Future investigations employing miRNA target validation assays and functional genomic studies are imperative to elucidate these mechanisms. Similarly, while our data suggests a potential role for IGF2BP1 and GCNT2 in HU- and Met-mediated γ-globin induction, the intricate molecular mechanisms governing their regulation and interaction within the γ-globin regulatory network necessitate further investigation. Specifically, the in-silico prediction of SP1 binding sites within the IGF2BP1 and FOXO3 promoters requires experimental validation through techniques such as chromatin immunoprecipitation (ChIP) assays. Moreover, further research is warranted to delineate the relative contributions of translational regulation by IGF2BP1 versus potential epigenetic modifications, including chromatin remodeling, on γ-globin gene expression. To comprehensively assess the temporal dynamics of HU and Met’s effects, future studies should extend beyond the current 24-hour time frame to evaluate the sustained impact of these agents on erythroid differentiation, γ-globin expression, and HbF production. These longitudinal investigations should also incorporate assessments of potential cytotoxic effects, compensatory mechanisms, and the synergistic or antagonistic interactions of HU and Met with other established HbF-inducing agents. Such studies are crucial for optimizing therapeutic strategies for hemoglobinopathies.

## Conclusion

This study elucidates the mechanisms by which HU and Met enhance γ-globin expression and HbF production in the K562 cell line. Our findings demonstrate that treatment with HU and Met significantly increases *IGF2BP1* expression while concurrently decreasing *GCNT2* levels, suggesting a regulatory pathway that facilitates γ-globin gene switching.

These insights contribute to a deeper understanding of the molecular dynamics involved in HbF regulation and highlight the potential of these drugs as therapeutic agents for disorders characterized by low HbF levels, such as sickle cell anemia and intermedia β-thal. By advancing our knowledge of γ-globin switching mechanisms, this research lays the groundwork for the development of targeted therapies aimed at improving patient outcomes in these debilitating diseases.

Overall, this study provides valuable insights into the molecular mechanisms of γ-globin regulation and emphasizes the potential of HU and 5-azacytidine (Met) as therapeutic agents in managing conditions characterized by inadequate HbF production. Future research should focus on further elucidating these pathways and exploring additional therapeutic options to enhance HbF levels in affected individuals.

## Electronic supplementary material

Below is the link to the electronic supplementary material.

Supplementary Material 1

## Data Availability

The raw data supporting the findings of this study will be made publicly available to qualified researchers without unreasonable restrictions. The public microarray dataset GSE90878, utilized in this analysis, is accessible for download from the National Center for Biotechnology Information Gene Expression Omnibus (NCBI GEO) database (http://www.ncbi.nlm.nih.gov/geo/). Additionally, all relevant experimental data, including gene expression levels for GCNT2, γ-globin, IGF2BP1, flow cytometry data, and associated microRNA expression profiles, will be provided upon request. The computational code and analytical scripts employed for the weighted gene co-expression network analysis (WGCNA) are also available upon request to facilitate further investigation.
