# Supplementary material for: The induction effect of hydroxyurea and metformin on fetal globin in the K562 cell line
Source: Mol Med. 2025 Apr 8;31:132. doi: 10.1186/s10020-025-01184-8 (PMC11978054; doi:10.1186/s10020-025-01184-8)

**Figure S1** Sample clustering to detect outliers. The color is proportional to the developmental stage (Red= fetal liver erythroblast and white=adult bone marrow erythroblast).


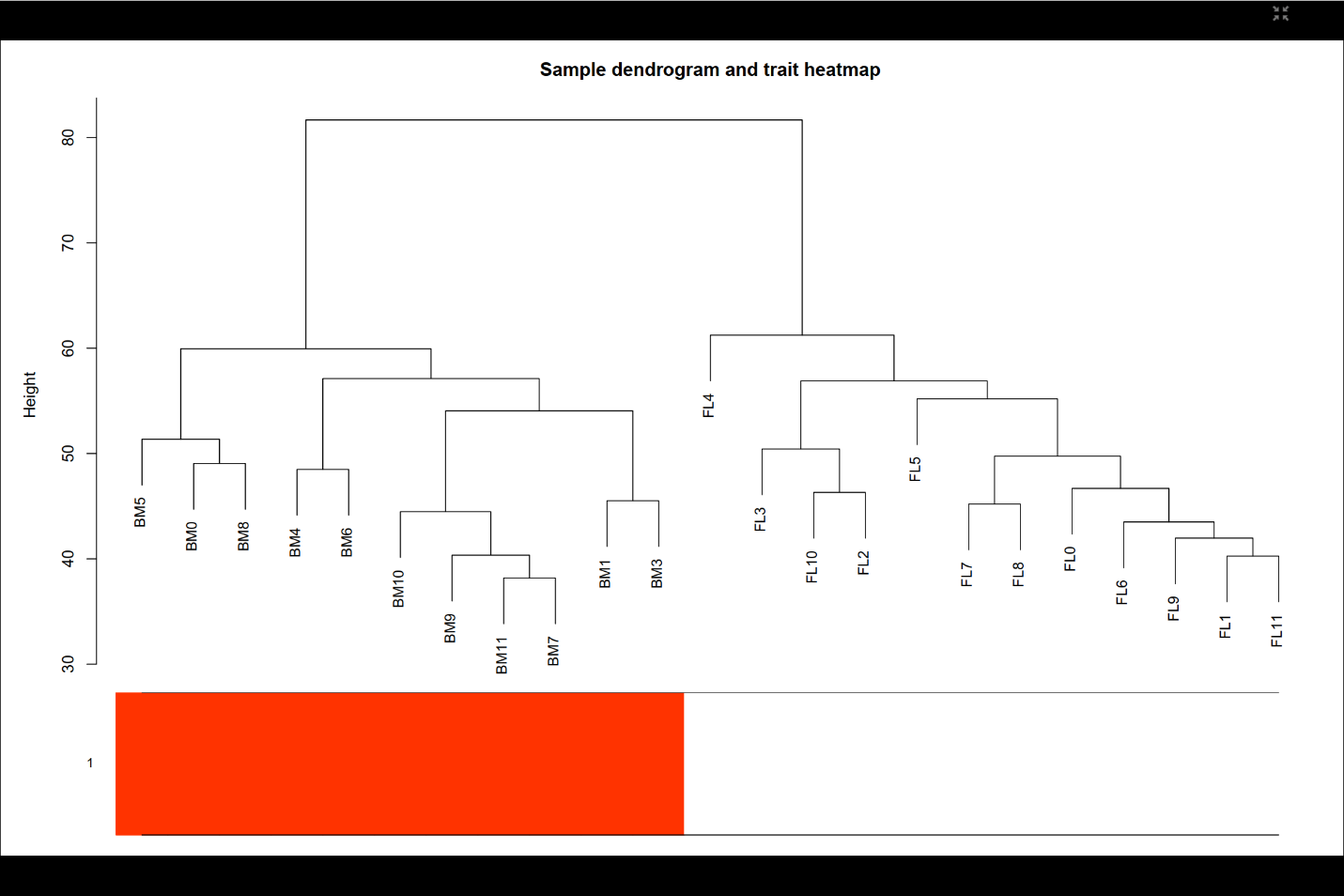


**Figure S2** Selection of the soft-thresholding powers. The left panel shows the scale-free fit index (y-axis) as a function of the soft-thresholding power (x-axis). The right panel displays the mean connectivity (degree, y-axis) as a function of the soft-thresholding power (x-axis). The power was set as 8, for the next analysis.


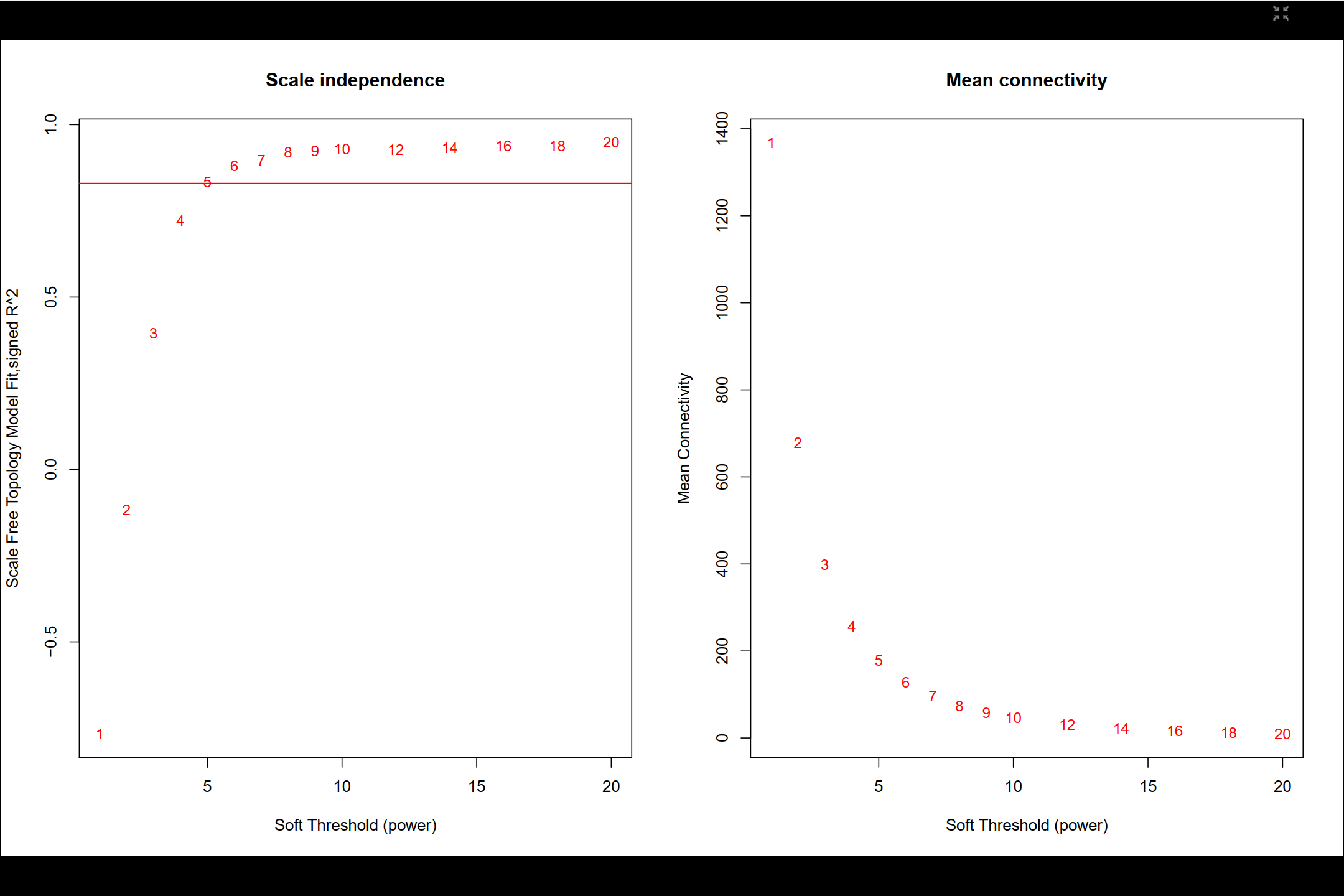


**Figure S3** Cluster dendrogram and module assignment from WGCNA. The branches correspond to highly interconnected groups of genes. Colors in the horizontal bar represent the modules


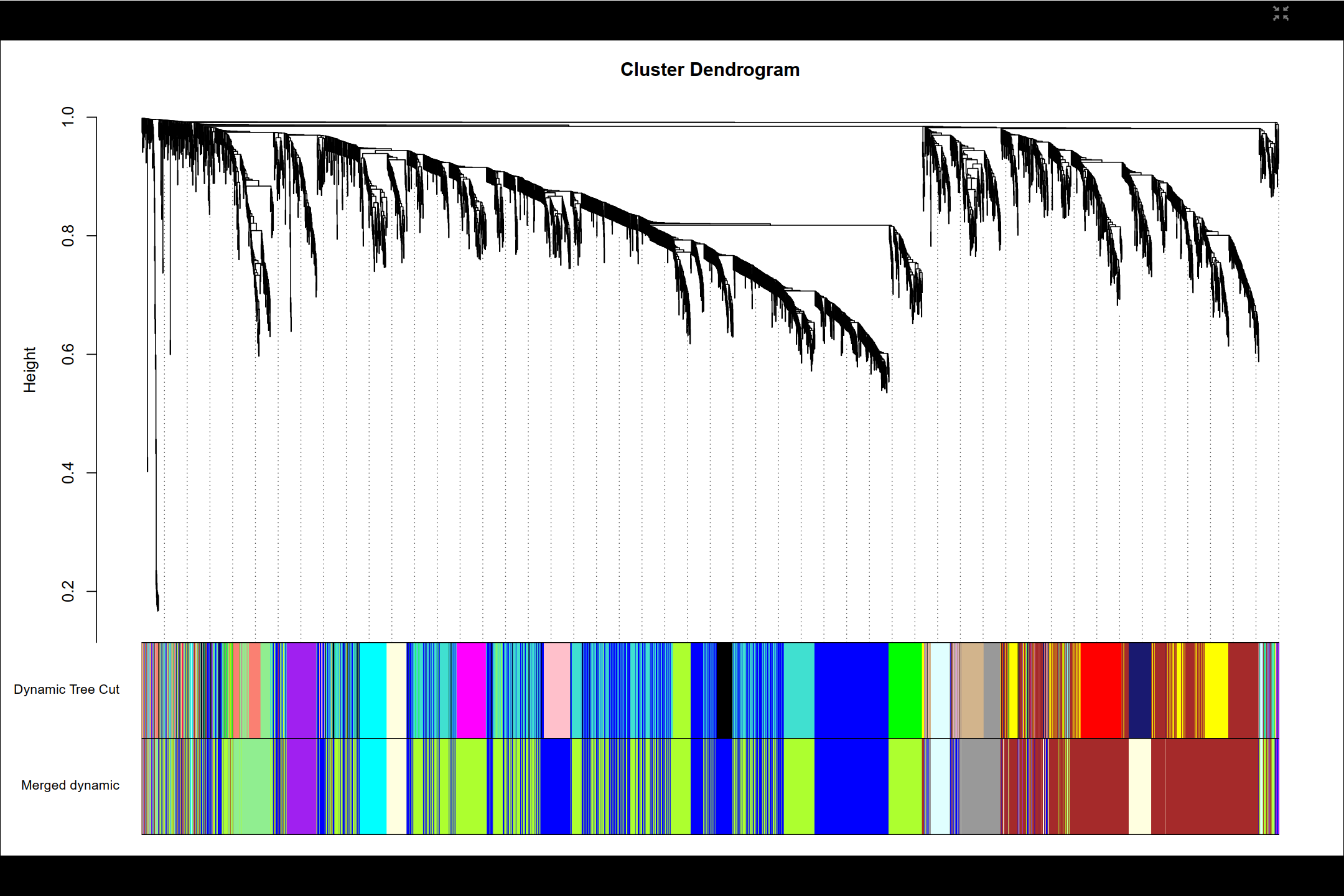


**Figure S4:** Selection of Best Hubgenes for reconstruction of co-expression network in Blue modules. Hubgenes with GS and MM > 0.9616 and 0.9556 respectively and fold changes |log_2_FC| ≥ 2 were selected


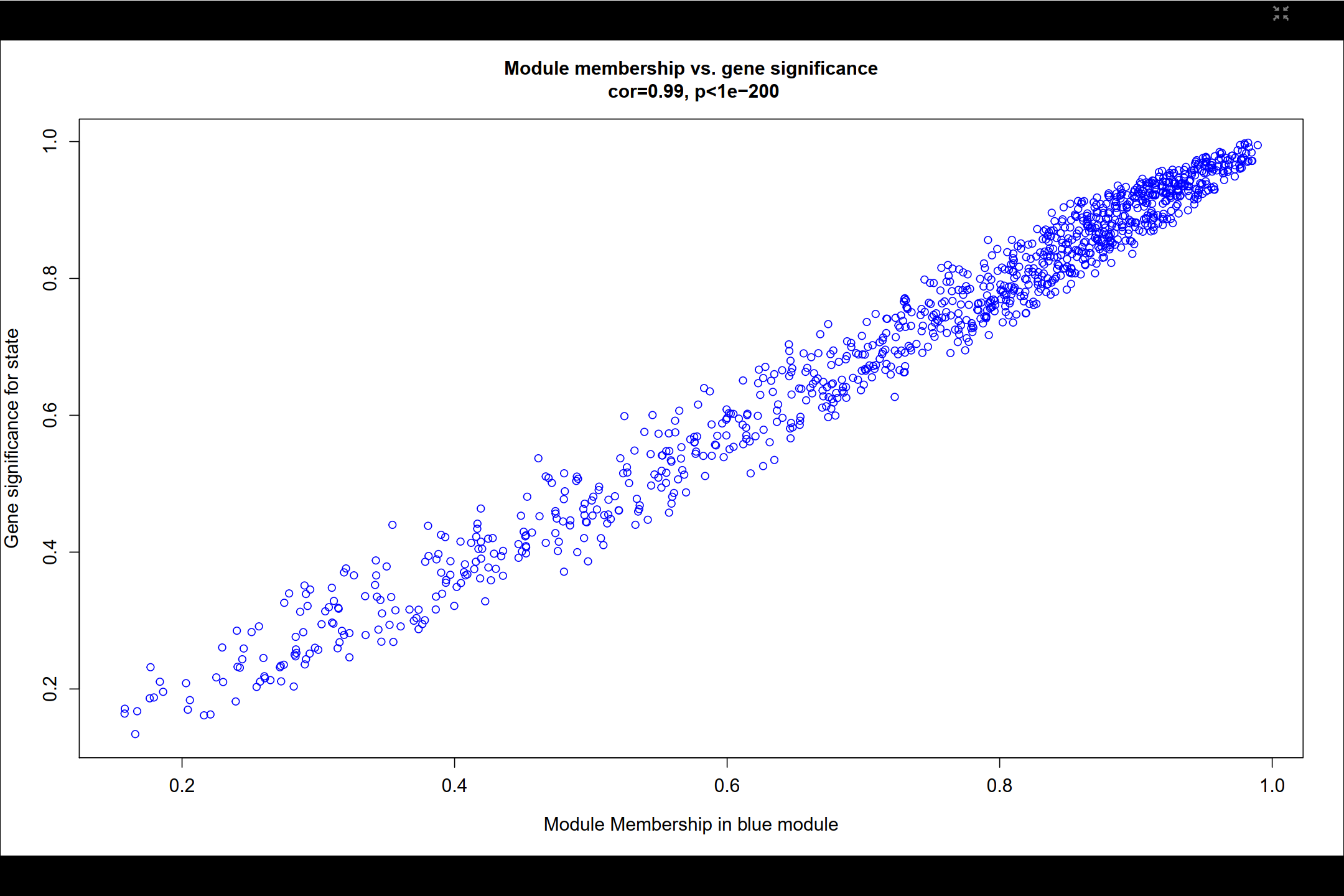


**Figure S5** Preservation of GSE90878 modules in comparison with GSE109186. Each module is represented by its color-code and name. Left figure indicate the preservation median rank. This factor is independent from module size and high median ranks correspond to low preservation. Right figure indicates Z_summary_ score. The dashed blue and black lines show the thresholds Z = 0 and Z = 2


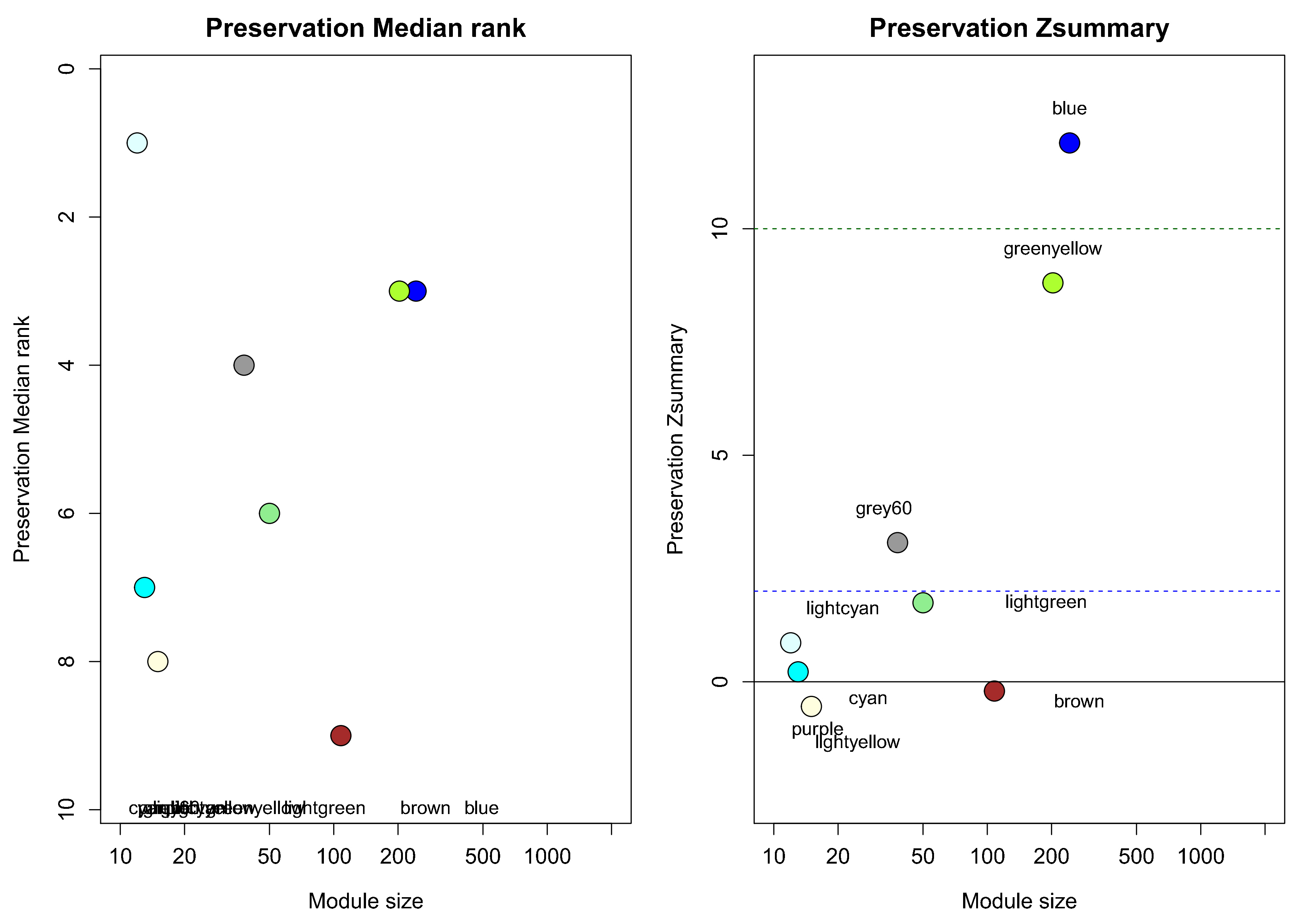

Supplement: Supplementary file 1 — Supplementary Material 1 [file 10020_2025_1184_MOESM1_ESM.docx]
